# Bending over backwards for soil emergence: KIPK and KIPK-LIKE1 regulate hypocotyl gravitropic growth

**DOI:** 10.1093/plcell/koaf073

**Published:** 2025-04-02

**Authors:** Gwendolyn K Kirschner

**Affiliations:** Assistant Features Editor, The Plant Cell, American Society of Plant Biologists; The James Hutton Institute, Invergowrie, Dundee DD2 5DA, UK

Charles Darwin observed that after germination, the uppermost parts of plant seedlings emerge from the soil in an arch-like shape (Darwin 1881). This arch is formed by the apical portion of the hypocotyl that bends downward, protecting the fragile tissues at the tip of the seedling as it grows through the soil (Fig. 1A). Following emergence, hypocotyl elongation is directed upward by negative gravitropism, where growth occurs against gravity so that the seedling can reach the sunlight (Jonsson et al. 2023).

**Figure 1.**
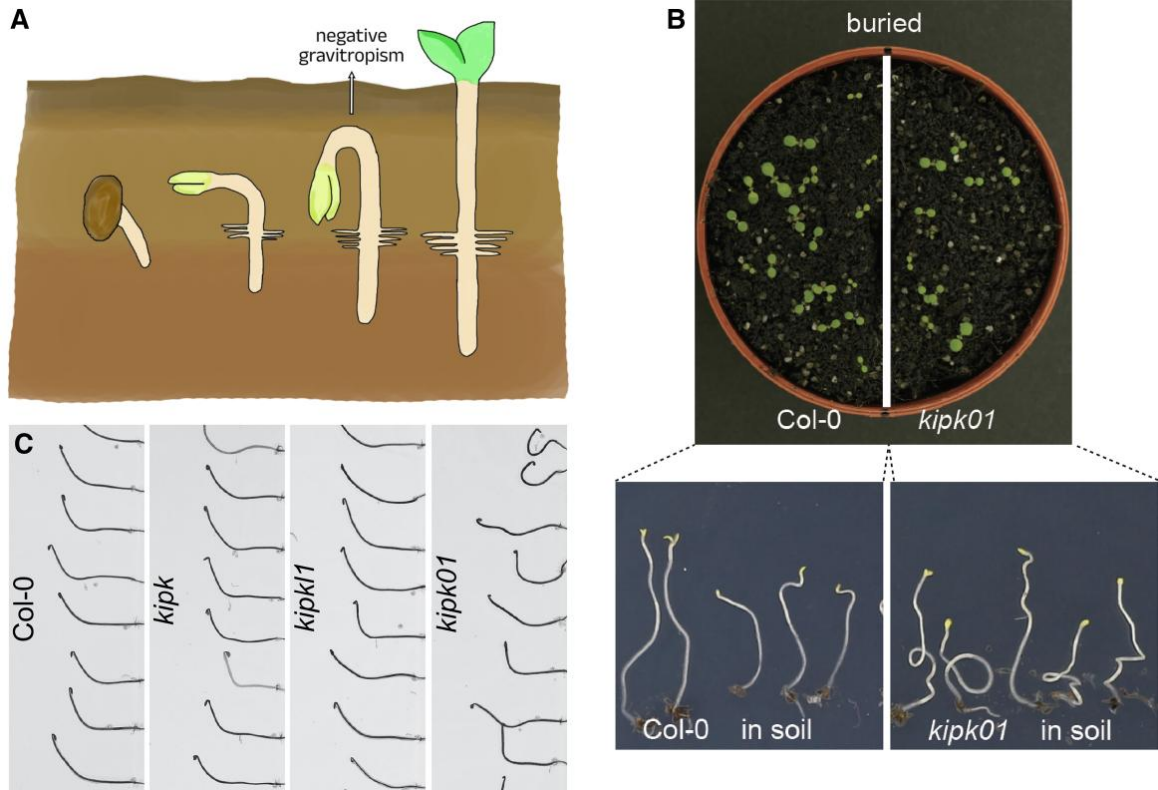
KIPK and KIPK-LIKE1 regulate hypocotyl bending. **A)** During seedling emergence from soil, the hypocotyl is bent into an apical hook to protect the fragile shoot tissues from mechanical injury. The hypocotyl then bends upward, driven by light and negative gravitropism, and breaks through the soil. **B)** Fewer *kipk kipkl1* double (*kipk01*) mutants are able to emerge through the soil compared with wild-type (Col-0) seedlings. **C)** Compared with wild-type (Col-0) dark-grown seedlings, *kipk kipkl1* double (*kipk01*) mutants bend more strongly after reorientation by 90°. **B, C)** Adapted from Xiao *et al*. (2025), Figure 3 and Figure 7.

The bending of the hypocotyl in response to gravity and directional light signals relies on an uneven distribution of the phytohormone auxin, which is regulated by polar auxin transport, particularly through the action of PIN-FORMED (PIN) auxin transporters (Jonsson et al. 2023). PINs are polarly distributed proteins in the plasma membrane, and their distribution is used to predict auxin transport streams in the plant. PIN proteins are activated by AGCVIII family kinases, plant-specific serine/threonine kinases that are characterized by an insertion between protein kinase subdomains VII and VIII. The AGC1 kinases D6 PROTEIN KINASE (D6PK) together with the related D6PK-LIKE1 (D6PKL1)–D6PKL3 promote auxin transport through the regulation of PINs during phototropic hypocotyl bending (Willige et al. 2013), but the biological functions of several other AGC1 kinases remain to be established.

**Yao Xiao and colleagues (Xiao et al. 2025)** analyze the function of the AGC1 kinases KINESIN-LIKE CALMODULIN-BINDING PROTEIN INTERACTING PROTEIN KINASE (KIPK) and its paralog KIPKL1 in gravitropic hypocotyl bending in Arabidopsis (*A. thaliana*). They find that the two proteins are required for efficient reorientation of seedling hypocotyls after they have been diverted from negative gravitropic growth through contact with soil particles. Consistently, fewer *kipk kipkl1* double mutant seedlings emerge from the soil when sown 1 cm buried in the soil (Fig. 1B), suggesting that controlled and dynamic gravitropic hypocotyl bending mediated by KIPK/KIPKL1 regulation is necessary for this process. *kipk kipkl1* double mutants also showed exaggerated negative gravitropism, that is, their hypocotyls bent more strongly than the wild type after dark-grown seedlings with elongated hypocotyls were reoriented by 90° (Fig. 1C).

Like PINs, KIPK and KIPKL1 are polarly distributed at, but not in, the basal (rootward) plasma membrane. This localization to the basal membranes appears to be mediated by interactions of the protein with phospholipids in the plasma membrane in a manner that is similar but not identical to the plasma membrane interaction of D6PK.

Differential distribution of PIN3 in the hypocotyl endodermis plasma membrane is reported to be crucial for gravitropic bending (Rakusová et al. 2011). Therefore, the authors tested the interplay between the KIPKs and auxin transport. KIPK and KIPKL1 were able to phosphorylate the PIN3 cytoplasmic loop and activate PIN3-mediated auxin efflux. *kipk kipkl1* double mutants displayed a strong reduction in basipetal auxin transport in hypocotyls of dark-grown seedlings. Even though KIPK protein localization did not change during hypocotyl bending, the *kipk kipkl1* double mutants showed differences in auxin accumulation. The auxin signaling reporter was expressed at a lower level in the hypocotyls of *kipk kipkl1* double mutants than in wildtype hypocotyls but at a higher level than wild type in the cotyledons. The expression of *KIPK* from an endodermis-specific promoter was sufficient to rescue the bending and auxin transport defects of *kipk kipkl1* double mutants. This suggests that *KIPK* and *KIPKL1* act redundantly to influence the cellular auxin response machinery.

In conclusion, Xiao and colleagues uncovered a previously unknown biological role of the KIPK and KIPKL1 proteins for the dynamic regulation of gravitropic hypocotyl bending.

**Recent related articles in *The Plant Cell*:**

(Xiong et al. 2023) show that the small ubiquitin-like modifier (SUMO) E3 ligase SAP AND MIZ1 DOMAIN-CONTAINING LIGASE1 (SIZ1) interacts with HOOKLESS1 (HLS1) in regulating apical hook development.

(Siao et al. 2023) analyze the role of the regulator of clathrin-mediated endocytosis ADAPTOR-ASSOCIATED PROTEIN KINASE1 (AAK1) for root tropic growth, including touch- and gravity-sensing responses.

(Li et al. 2024) identify 2 nonspecific phospholipase C enzymes that control the asymmetric distribution of phosphatidic acid and thereby modulate auxin-controlled plant growth and tropic responses.

## Data Availability

No new data were generated or analysed in support of this.

## Dive Curated Terms

The following phenotypic, genotypic, and functional terms are of significance to the work described in this paper:

PIN3 Gramene: AT1G70940
PIN3 Araport: AT1G70940

MIZ1 Gramene: AT2G41660
MIZ1 Araport: AT2G41660
